# Hypertension Prevalence, Awareness, Treatment and Control, and Associated Factors: Results from a National Survey, Jordan

**DOI:** 10.4061/2011/828797

**Published:** 2011-12-01

**Authors:** H. Y. Jaddou, A. M. Batieha, Y. S. Khader, A. H. Kanaan, M. S. El-Khateeb, K. M. Ajlouni

**Affiliations:** ^1^Department of Community Medicine, Faculty of Medicine, Jordan University of Science and Technology, Irbid 22110, Jordan; ^2^Department of Cardiology, Rush University Medical Center, 600 South Paulina Street, Chicago, IL 60612, USA; ^3^National Center for Diabetes, Endocrinology and Genetics, P.O. Box 13165, Amman 11942, Jordan

## Abstract

The study examined prevalence, awareness, treatment and control of hypertension (HTN), and associated factors and to evaluate the trend in hypertension between 2009 (period 2) and 1994–1998 (period 1). A national sample of 4117 adults aged 25 years and older was selected. Prevalence rate of HTN (SBP ≥ 140 or DBP ≥ 90 or on antihypertensive therapy) was 32.3% and was higher than the 29.4% prevalence rate reported in period 1. Prevalence rate was significantly higher among males, older age groups, least educated, obese, and diabetics than their counterparts. The rate of awareness among hypertensives was 56.1% and was higher than the 38.8% rate reported form period 1 data. Awareness was positively associated with age, smoking, and diabetes for both men and women, and with level of education and body mass index for men. Rate of treatment for HTN among aware patients was 63.3% and was significantly higher than the 52.8% rate reported in period1. Control rate of HTN among treated hypertensives was 39.6%; significantly higher than the 27.9% control rate in period 1. Control of HTN was positively associated with age but only for women. In conclusion, HTN is still on the rise in Jordan, and levels of awareness and control are below the optimal levels.

## 1. Introduction

HTN is a major public health problem of worldwide distribution and is the most common cardiovascular disease (CVD) risk factor [1]. It is responsible for one half of coronary heart disease (CHD) and about two thirds of cerebrovascular accidents [2]. By 2030, 23 million cardiovascular deaths are projected to have HTN, with about 85% occurring in low and middle-income countries [1]. Research published between 1980 and 2002 indicate the prevalence of HTN in developing countries increased at a higher rate than in developed countries [3]. Prevention of HTN is possible, and early detection and treatment can reduce the incidence of complications including stroke, CHD, heart failure, and kidney disease [4], and yet the levels of control of hypertension are low worldwide. Economically developed countries have higher rates of HTN than in developing countries [5]. However, data reported in the last decade indicate that the prevalence, awareness, treatment, and control of hypertension in economically developing countries are coming closer to those in economically developed countries [6].

Recent epidemiological studies on prevalence, awareness, treatment, and control of HTN in Jordan are scarce. The few community-based studies [7, 8] conducted between 1994 and 1996 in Jordan demonstrated a 16.1 and 16.3% prevalence rates of HTN (cut-off point 160/90 mm Hg) with concomitant low levels of awareness, treatment, and control. Since that time, several activities have been implemented to face this challenge in HTN which have not been evaluated. Moreover, the last decades showed a remarkable improvement in treatment of hypertension due to introduction of new antihypertensive medications and the development of international guidelines for detection and management [9, 10] 

This paper was conducted to assess the prevalence, awareness, treatment, and control of HTN using data from a 2009 national population survey and to examine the trend in HTN in Jordan by comparing these data with a pooled community-based data collected during the period between 1994 and 1998 in Jordan.

## 2. Materials and Methods

This study is a subset of a national population-based household survey that was conducted during the period between July 1st and November 30, 2009. Details of the sampling and data collection were reported elsewhere [11, 12]. Briefly, a complex multistage sampling technique was used to select the households from the 12 governorates of Jordan. Each of the selected households was visited by members of the study team, and, after been informed of the purpose of the study, all members aged ≥7 years were invited to report to the health center in their catchment area centre in the next day after an overnight fast. Subjects on regular medications were asked not to bring all their medications with them to the survey site. The present study deals exclusively with adults aged ≥25 years. 

Interview questionnaire was administered by trained interviewers and gathered information and specific history on HTN, DM, lipids, smoking behavior, and other socio-demographic characteristics. Anthropometric measurements including weight, height were measured with the subjects wearing light clothing and no shoes. Body mass index (BMI) was calculated as the ratio of weight in kilograms to the square of height in meters. BP was measured using standardized mercury sphygmomanometer with the appropriate cuff that covers two-thirds of the upper arm. A physician or trained nurse performed the BP measurement with the subject being in the sitting position, the arm at the level of the heart, and after 5 minutes rest. The cuff was inflated at a rate of 2-3 mm Hg per second. Systolic blood pressure (SBP) was taken upon hearing the first sound, and diastolic blood pressure (DBP) was taken upon complete disappearance of Korotkoff sounds (phase V). HTN was defined as SBP ≥ 140 mm Hg and/or DBP ≥ 90 mm Hg and/or use of antihypertensive medication. Hypertensive subjects were considered aware of HTN when having been told by a physician during the last year that they have hypertension or when report taking antihypertensive medication. Participants were considered to be treated for HTN when report using antihypertensive medication. HTN control was defined as SBP < 140 mm Hg and DBP < 90 mm Hg among treated patients. Body mass Index (BMI) was calculated by dividing the weight in kilogram on the height in meters squared. Participants with BMI of 30 kg/m^2^ or more were considered obese, while those with BMI values that range between 25 kg/m^2^ and <30 kg/m^2^ were considered overweight. HTN prevalence was age-sex adjusted by direct standardization to the Jordan 2004 standard population.

To examine trends in prevalence, awareness, and control of hypertension we pooled data from previously published [7, 8, 13] and unpublished population-based surveys conducted over the 1994–1998 period. The primary focus of those surveys was on cardiovascular disease risk factors among adults 25 years of age and older. Details of the sampling and data collection of those surveys are presented elsewhere [7, 8, 13]. Briefly, a systematic sampling technique was used to select the households from six communities located in the North, South, Middle, and East of Jordan and of approximately 80,000 inhabitants. The procedures used in those surveys, including training of study teams, home visits, invitation of eligible participants, consenting, settings (primary health care centers of Ministry of Health), interview questionnaire, verification of antihypertensive medications, application of physical measures of blood pressure, weight, and height were identical to the procedures used in the 2009 study. A total of 3905 individuals who participated in those surveys were included in the analysis with a response rate ranging between 45% and 78%.

## 3. Statistical Analysis

Statistical analyses were performed using the Statistical Package for Social Sciences software, SPSS (SPSS Inc., Chicago, Ill, USA) version 16. Frequency distribution was used to describe the dichotomous variable of hypertension prevalence, awareness, treatment, and control with the other categories of participants' characteristics. Bivariate analyses were performed to test for independent distribution of participants' characteristics among levels of hypertension using *χ* ^2^ test. The odds ratios (OR) and their *P* values were estimated by performing multivariate logistic regression models to test the association between hypertension and certain sociodemographic, behavioral, and health characteristics of participants. 

To examine the trend in hypertension, data on 3905 participants gathered during the period 1 were compared with period 2 data. Bivariate comparisons were performed to test for the independent distribution of hypertension and other participants' characteristics by study period using *χ* ^2^ test. A *P* value of ≤0.05 was considered statistically significant.

## 4. Results

In 2009, a total of 4117 eligible individuals were included in the analysis, with a response rate of 68%. The main reasons behind nonparticipation were inability to leave from work or to remain fasting prior to blood tests. As with period 1 surveys, the response rate was significantly higher among women than among men, dictating the need for sex-specific analysis of data. 

As shown in Table 1, about one-third (30.5%) of the sample were 50 years of age and older, 25.4% were males, 43.6% were either illiterate or with <High School (HS) diploma, 32.2% were holding college diploma or higher, 45.5% have a family monthly income of <500 US$, and 86.3% have <850 US$ per month. Table 1 also shows that 16% were current smokers, 35.3% were overweight, 45.1% obese, 16.4% diabetics, and 54.2% had high levels of serum cholesterol. 

Of the 4117 participants, a total of 1329 (32.2%) were categorized as having HTN. Table 1 indicates that the prevalence rate of HTN is significantly higher among males and the older age groups with a sharp rise in the 50 years and older age groups. Participants with a lower level of education and income, obesity, DM, and hypercholesterolemia have significantly higher rates of HTN than their counterparts. The findings from sex-specific multiple logistic regression analysis indicate that increasing age and BMI, the presence of DM, and the lowest level of education were independently significantly associated with HTN, but no evidence of a significant association with income, smoking, and high serum cholesterol was found for either men or women (Table 2). For men, the risk of having HTN for the age groups 50–64 years and 65+ years is 3.5 and 7.2 times, respectively, compared to the youngest age group, while the risk of HTN for women in the same age groups is 7.3 and 14.1 times, respectively, compared to the youngest age group (Table 2). 

Of the 1329 hypertensive 2009 participants, 746 (56.1%) were aware of having hypertension. As indicated in Table 1, rate of awareness significantly increases with age, BMI, lowest level of education, marriage, and presence of DM. Results of sex-specific multiple logistic regression analysis are depicted in Table 2. For men, awareness independently correlates positively with the older age group, highest level of education, smoking, obesity, DM, and hypercholesterolemia; no association was detected with marital status or income. For women, awareness independently correlates positively with age, smoking, and DM; no association was detected with education, marital status, income, BMI, or hypercholesterolemia. 

Of the 746 hypertensive individuals aware of having HTN, 472 (63.3%) were on antihypertensive treatment (Table 1). Older patients, males, nonsmokers, and patients with hypercholesterolemia are significantly more likely to receive treatment for HTN than their counterparts. Sex-specific multiple logistic regression analysis indicates that increased age for men and women and income for women were significantly associated with receiving treatment for HTN; no significant association was found with educational attainment, smoking, BMI, DM, and hypercholesterolemia (Table 3). 

Among the 472 of the 2009 hypertensive participants who were on antihypertensive medications, 187 (39.6) had controlled HTN. Univariate analysis shows that older, more educated, more affluent, and smokers have significantly higher rates of controlled HTN than their counterparts (Table 1). The findings from sex-specific multivariate logistic regression analysis indicated that only women in the age groups 50 years and older were significantly more likely to have controlled HTN than those in the age group 25–34 years; no significant association was found with education, smoking, BMI, DM, and hypercholesterolemia for both men and women (Table 3).

To examine the trend in HTN prevalence, awareness, treatment, and control among Jordanian adults, we compared the 1994–1998 (period 1) data with the 2009 (period 2) data. Bivariate analysis show no significant differences in age and gender of the participants in the two periods but BMI was significantly higher among period 2 participants (*P* value =  .00) (Table 4). As indicated in Table 4, prevalence rate of HTN had increased from 29.4% in period 1 to 32.3% in period 2 (*P* value =  .00) (31.2% versus 35.5% for men, and 28.3% versus 31.2% for women). Data also show that awareness of HTN in period 2 increased from 38.8% to 56.1% in period 2 (*P* value =  .00) (31.7% versus 54.5% for men, and 45.9% versus 57.7% for women). The rate of antihypertensive therapy among aware patients increased from 52.8% in period 1 to 63.3% in period 2 (*P* value =  .00) (52.5% versus 87.4% for men, and 53% versus 83.4% for women). Among patients receiving antihypertensive treatment, the control rate of HTN increased from 27.9% in period 1 to 39.6% in period 2 (*P* value =  .00) (28.3% versus 44.4% for men, and 27.5% versus 38.2% for women).

## 5. Discussion

This is the first national survey conducted to examine the prevalence, awareness, treatment, and control of HTN and their associated factors among Jordanian adults. 

Study data indicate that about one-third of adult Jordanians aged 25 years and older are afflicted with HTN. The 32.2% prevalence rate of HTN reported in this study is in the upper range of the 26.1% −32.1% figures reported from other Arab countries [14–17], lower than the 42%–44.9% rate reported in Eastern Europe [18, 19], the 36%–55% rates reported in other western European countries [20–22], and the 35.8% rate reported in Australia [23], but is higher than the 27%-28% rates reported in China [24], Canada, and the United States [19]. Variations in prevalence rates of HTN by regions could be attributed to several factors including changes in lifestyle associated with urbanization, ethnic differences, nutritional status, birth weight [25], variations in study designs, the age range included in the study, the time period of the study, and the type of blood pressure machines used. The high prevalence rate of HTN in Jordan could be partially attributed to the epidemic of obesity among Jordanians, adoption of western lifestyle [26], and to the fact that >80% of the population lives in urban areas. Moreover, the population of Jordan has witnessed extremely stressful conditions, starting with the exodus of Palestinians to Jordan during the 1948 Arab-Israeli war, followed by another wave of migration of masses of Palestinians from the West Bank of Jordan to Jordan during 1967 Arab-Israeli war, and by a third wave of immigration of approximately 350,000 Jordanians (about 9% of the total population of Jordan at that time) from the Arabian Gulf States to Jordan during the 1991 Gulf War. These conditions subjected the Jordanian population to the full blown ramifications of social, psychological, and economic stress that could have increased the rates of mental and physical illness, including HTN.

The prevalence rate of HTN in 2009 was significantly higher than it was in the 1994–1998 period. This difference in HTN prevalence remained significant even after controlling for the effect of BMI (data not reported). This trend is in line with the secular increase in the HTN prevalence in economically developing countries [26–28]. Consistent with the findings from several studies, HTN was positively associated with male gender [23, 29] and age [7, 13, 14]. However, in older age groups, the risk of having HTN rises more dramatically among females than males, especially after the age of 50 years. In their study of trends in hypertension in the MONICA project, Gasse et al. [30] concluded that “… in advanced age the increase in the rate of hypertension is steeper in women than in men.” In line with the findings from other research, HTN is positively associated with BMI [29]. Moreover, study data show that overweight and obese men are at higher risk of developing HTN than women of the same weight categories, a finding that is supported in the literature [31, 32]. Our study indicates that HTN is inversely associated with level of education [33, 34]. Highly educated people are usually well informed about health issues including HTN and more prone to adopt healthy lifestyle habits such as a healthy diet, exercise, and the maintenance of an ideal body weight. Moreover, educated people maintain a greater sense of control over their lives and a tend to have a greater level of social support than those with a lower level of education [35]. Data in this study shows that HTN is positively associated with DM, a finding that is highly supported in the research literature [36]. Moreover, the odds ratio of HTN was higher in diabetic women than in diabetic men. To our knowledge, there is no existing research examining the sex differences in HTN among diabetics. However, meta-analysis studies [37, 38] indicate that the odds of CHD mortality were significantly higher among diabetic women, as compared with diabetic men.

Compared to period 1 data, the rate of awareness of HTN has increased by 45% in period 2 (38.8% for men versus 56.1% for women). Despite the fact that the 56.1% awareness rate of HTN in Jordan is higher than the rates reported from other countries [25, 29, 36], it is still far below than the 63%–82.5% rates reported from developed countries [2, 20, 39]. Awareness in this study is positively associated with female gender. This finding is strongly supported in the research literature [2, 20, 23, 40]. It appears from this study that age is positively associated with awareness of HTN among women but only starts to show a positive effect after the age of 65 for men. On the other hand, awareness was positively associated with the highest level of education for men, whereas no such association was found for women. Coexistence of other CVD risk factors (smoking, obesity, DM, and high levels of serum cholesterol) is positively associated with awareness of HTN for men, but only DM and smoking are positively associated with awareness for women. People have recently become more aware of CVD risk factors, and the presence of one or more risk factors increases the tendency for those patients and/or their treating physicians to check for the presence of other risk factors, including HTN. The absence of association between awareness of HTN and obesity and high serum cholesterol among women is perhaps related to the fact that the illiteracy rate is >3 times higher among women than men (12.5% versus 3.8%), thus decreasing the likelihood that women will be better informed of the association between those CVD risk factors with HTN.

The present study indicates that the rate of treatment of HTN among those aware of having HTN increased from 52.4% in period 1 to 63.3% in period 2. This rate is comparable to the 61.4%–82.5% treatment rates reported from developed countries [2, 3, 20, 36, 39]. In line with findings from other studies [3, 36], the rate of treatment is positively associated with age. Older subjects are more inclined to be compliant with the medications prescribed for them [36]. 

Data in this study show that the rate of control of HTN among those receiving antihypertensive medications has increased from 27.9% in period 1 to 39.6% in period 2. This rate is comparable to the 35.1%–66.4% control rates reported from economically developed countries [1, 2, 23, 39]. 

In conclusion, it is evident that hypertension is a common public health problem in Jordan and is still on the rise. Awareness, treatment, and control of HTN have shown considerable improvement over time but are still below the optimal levels.

## Figures and Tables

**Table 1:** Hypertension prevalence, awareness, treatment, and control by sociodemographic and health variables of the 2009 survey (*N* = 4117)*.

Variables	*N* (%)	Prevalence, *n *(%)	Awareness, *n *(%)	Treatment, *n *(%)	Control, *n *(%)
Overall	4117	1329 (32.3)	746 (56.1)	472 (63.3)	187 (39.6)
Age		.00	.00	.00	.00
25–34	964 (23.4)	95 (9.9)	31 (32.6)	12 (38.7)	8 (68.8)
35–49	1899 (46.1)	460 (24.2)	208 (45.2)	118 (57.2)	57 (47.5)
50–64	962 (23.4)	563 (58.5)	358 (63.6)	241 (67.3)	91 (38.2)
65+	292 (7.1)	211 (72.3)	149 (70.6)	101 (76.8)	31 (30.1)
Sex		.01	.52	.01	.27
Men	1045 (25.4)	371 (35.5)	203 (54.7)	136 (67)	58 (42.9)
Women	3072 (74.6)	958 (31.2)	543 (56.7)	336 (61.9)	129 (38.2)
Education		.00	.02	.78	.01
<high school	1781 (43.6)	793 (44.5)	469 (59.1)	298 (63.5)	109 (36.6)
High school	991 (24.2)	249 (25.1)	125 (50.2)	77 (61.6)	30 (38.5)
>high school	1315 (32.2)	270 (20.5)	142 (52.6)	90 (63.4)	48 (52.9)
Marital status		.00	.00	.29	.00
Single	351 (8.5)	54 (15.4)	17 (31.5)	10 (58.8)	8 (84.6)
Married	3766 (91.5)	1275 (33.9)	729 (57.2)	462 (63.4)	179 (38.6)
Income (US$)**		.00	.25	.28	.00
<500	1848 (45.5)	704 (38.1)	409 (58.1)	253 (61.9)	81 (32.2)
500–850	1659 (40.8)	467 (28.1)	254 (54.4)	165 (65)	79 (48.0)
>850	558 (13.7)	143 (25.6)	74 (51.7)	48 (64.9)	24 (50.8)
Current smoking		.00	.08	.05	.04
No	3457 (84.0)	1159 (33.5)	640 (55.2)	410 (64.1)	156 (38.0)
Yes	660 (16.0)	170 (25.8)	106 (62.4)	62 (58.5)	31 (50.0)
BMI, kg/m^2^		.00	.01	.38	.1
<25	808 (19.6)	92 (11.4)	39 (42.4)	24 (61.5)	11 (43.8)
25–29	1453 (35.3)	369 (25.4)	199 (53.9)	122 (61.3)	56 (46.1)
30+	1856 (45.1)	868 (46.8)	508 (58.5)	326 (64.2)	120 (36.8)
DM		.00	.00	.4	.35
Absent	3442 (83.6)	681 (28.1)	310 (45.5)	198 (63.9)	78 (39.3)
Present	675 (16.4)	400 (61.8)	294 (73.5)	193 (65.6)	68 (35.4)
High cholesterol		.00	.96	.01	.23
Absent	1887 (45.8)	505 (26.8)	283 (56.0)	170 (60.1)	72 (42.6)
Present	2230 (54.2)	824 (37.0)	463 (56.2)	302 (65.2)	114 (37.8)

*Values on certain variables did not add up to 100% due to missing data.

**Income: monthly income.

**Table 2:** Adjusted odds ratio (OR) and their *P* values of predictors of hypertension prevalence and awareness by sex.

	Prevalence				Awareness			
Variables	Men		Women		Men		Women	
	OR	*P* value	OR	*P* value	OR	*P* value	OR	*P* value
Age								
25–34	1		1		1		1	
35–49	1.17	.59	2.32	.00	.65	.43	1.94	.03
50–64	3.55	.00	7.34	.00	2.25	.13	3.05	.00
65+	7.18	.00	14.1	.00	3.61	.03	4.37	.00
Education								
<high school	1		1		1		1	
High school	.84	.35	.84	.15	1.24	.5	1.12	.57
>high school	.66	.04	.69	.01	2.13	.02	1.19	.44
Marital status								
Single	1		1		1		1	
Married	1.04	.92	1.4	.09	1.84	.47	2.21	.89
Income**								
<300	1		1		1		1	
300–599	1.28	.15	.82	.07	.83	.49	.94	.71
600+	1.16	.56	.99	.94	.48	.08	1	.99
Current smoking								
No	1		1		1		1	
Yes	.9	.23	.91	.21	2.27	.00	1.83	.02
BMI, kg/m^2^								
<25	1		1		1		1	
25–29	2.72	.00	1.7	.00	1.75	.21	1.21	.55
30+	5.52	.00	3.74	.00	2.78	.02	1.33	.35
DM								
Absent	1		1		1		1	
Present	1.63	.02	2.57	.00	1.98	.02	3.56	.00
High cholesterol								
Absent	1		1		1		1	
Present	1.21	.21	1.05	.65	.54	.02	1.02	.89

**Table 3:** Adjusted odds ratio (OR) and their *P* values of predictors of hypertension treatment and control by sex.

	Treatment				Control			
Variables	Men		Women		Men		Women	
	OR	*P* value	OR	*P* value	OR	*P* value	OR	*P* value
Age								
25–34	1		1		1		1	
35–49	7.82	.02	1.64	NS	.64	NS	3.44	NS
50–64	64.78	.00	4.12	.01	.95	NS	5.24	.05
65+	54.98	.00	5.2	.01	.86	NS	10.0	.01
Income**								
<300	1		1		1		1	
300–599	.69	NS	1.97	.03	.62	NS	.66	NS
600+	.61	NS	2.3	.05	.73	NS	.78	NS

Education, marital status, smoking, BMI, DM, and High cholesterol variables were included in the treatment and control models and were statistically not significant.

**Table 4:** Distribution of prevalence, awareness, treatment and control of hypertension, and sociodemographic characteristics of participants by study period.

Variable	Period 1		Period 2		*P* value
	N	%	N	%	
Age					
<40	1619	(41.5)	1626	(39.5)	
40+	2286	(58.5)	2491	(60,5)	.1
Sex					
Male	1545	(39.1)	1548	(37.6)	
Female	2360	(60.9)	2569	(62.4)	.1
BMI					
<25	957	(24.5)	701	(17)	
25–29.9	1335	(34.2)	1392	(33.8)	
30+	1613	(41.3)	2024	(49.2)	.00
Prevalence	1148	(29.4)	1329	(32.3)	.00
Awareness	445	(38.8)	746	(56.1)	.00
Treatment	234	(52.8)	472	(63.3)	.00
Control	65	(27.9)	187	(39.6)	.00
